# Crystal structure of poly[μ_6_-adipato-di­aquadi-μ_2_-oxalato-didysprosium(III)]

**DOI:** 10.1107/S1600536814024544

**Published:** 2014-11-15

**Authors:** Zhi-Feng Li, Yi-Chao Zhang, Xiao-Qin Hu, Chun-Xiang Wang

**Affiliations:** a School of Materials Science and Engineering, Jiangxi University of Science and Technology, Ganzhou 341000, People’s Republic of China

**Keywords:** crystal structure, dysprosium(III) complex, three-dimensional coordination polymer, oxalate, adipate

## Abstract

In the title coordination polymer, [Dy_2_(C_6_H_8_O_4_)(C_2_O_4_)_2_(H_2_O)_2_]_*n*_, the asymmetric unit consists of one Dy^3+^ cation, one half of an adipate anion, two halves of oxalate anions and one coordinating water mol­ecule. The adipate and oxalate ions are located on centres of inversion. The Dy^3+^ cation has a distorted tricapped trigonal–prismatic geometry and is coordinated by nine O atoms, four belonging to three adipate anions, four to two oxalate anions and one from an aqua ligand. The cations are bridged by adipate ligands, generating a two-dimensional network parallel to (010). This network is further extended into three dimensions by coordination of the rigid oxalate ligands and is further consolidated by O—H⋯O hydrogen bonds. A part of the adipate anion is disordered over two positions in a 0.75:0.25 ratio.

## Related literature   

For the isotypic structures of La, Sm and Gd complexes, see: Dan *et al.* (2005 ▶); Li & Wang (2010 ▶); Li (2011 ▶).
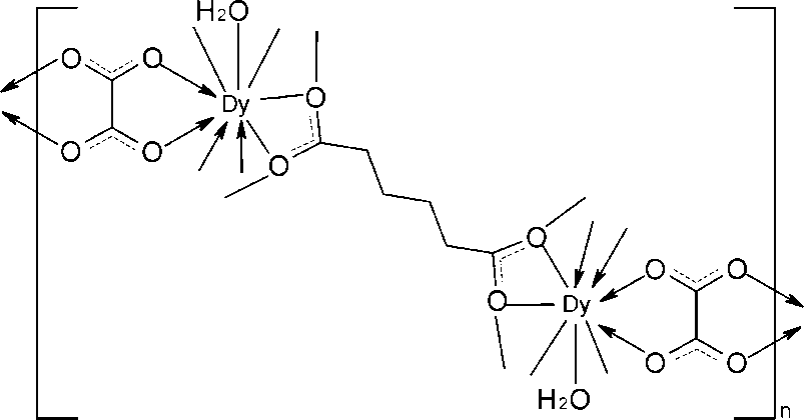


## Experimental   

### Crystal data  

[Dy_2_(C_6_H_8_O_4_)(C_2_O_4_)_2_(H_2_O)_2_]
*M* *_r_* = 681.20
Triclinic, 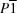

*a* = 6.772 (2) Å
*b* = 6.929 (2) Å
*c* = 8.949 (3) Å
α = 104.916 (5)°
β = 108.069 (4)°
γ = 104.306 (4)°
*V* = 360.5 (2) Å^3^
*Z* = 1
Mo *K*α radiation
μ = 10.37 mm^−1^
*T* = 295 K
0.21 × 0.09 × 0.07 mm

### Data collection  

Bruker SMART APEXII CCD area-detector diffractometer
Absorption correction: multi-scan (*SADABS*; Sheldrick, 2003 ▶) *T*_min_ = 0.245, *T*_max_ = 0.531
1813 measured reflections
1234 independent reflections
1179 reflections with *I* > 2σ(*I*)
*R*_int_ = 0.014

### Refinement  

*R*[*F* ^2^ > 2σ(*F* ^2^)] = 0.021
*wR*(*F* ^2^) = 0.057
*S* = 1.05
1234 reflections
122 parameters
H-atom parameters constrained
Δρ_max_ = 1.41 e Å^−3^
Δρ_min_ = −1.45 e Å^−3^

### 

Data collection: *APEX2* (Bruker, 2004 ▶); cell refinement: *SAINT* (Bruker, 2004 ▶); data reduction: *SAINT*; program(s) used to solve structure: *SHELXS97* (Sheldrick, 2008 ▶); program(s) used to refine structure: *SHELXL97* (Sheldrick, 2008 ▶); molecular graphics: *SHELXTL* (Sheldrick, 2008 ▶); software used to prepare material for publication: *SHELXTL*.

## Supplementary Material

Crystal structure: contains datablock(s) I, New_Global_Publ_Block. DOI: 10.1107/S1600536814024544/gk2622sup1.cif

**Fig. 1.**
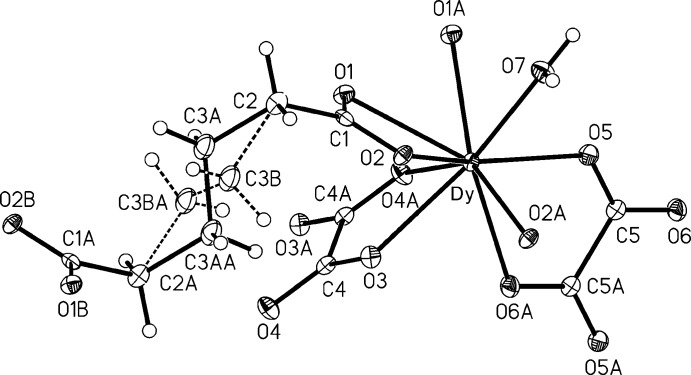
The fragment of the structure of the title compounds, with the atom-numbering scheme. Displacement ellipsoids are drawn at the 30% probability level and H atoms are shown as small spheres of arbitrary radii. Symmetry code: (i) -x, 1 - y, 1 - z; (ii) 1 - x, 1 - y, 1 - z; (iii) -x, - y, 1- z; (iv) - x, - y, -z; (v) 1 - x, 1 - y, 2 - z.

Structure factors: contains datablock(s) I. DOI: 10.1107/S1600536814024544/gk2622Isup2.hkl

x y z x y z x y - z x y z x y z . DOI: 10.1107/S1600536814024544/gk2622fig1.tif
The fragment of the structure of the title compounds, with the atom-numbering scheme. Displacement ellipsoids are drawn at the 30% probability level and H atoms are shown as small spheres of arbitrary radii. Symmetry code: (i) −*x*, 1 − *y*, 1 − *z*; (ii) 1 − *x*, 1 − *y*, 1 − *z*; (iii) −*x*, − *y*, 1*- z*; (iv) − *x*, − *y*, −*z*; (v) 1 − *x*, 1 − *y*, 2 − *z*.

CCDC reference: 1033325

Additional supporting information:  crystallographic information; 3D view; checkCIF report

## Figures and Tables

**Table 1:** Hydrogen-bond geometry (, )

*D*H*A*	*D*H	H*A*	*D* *A*	*D*H*A*
O7H7*A*O3^i^	0.85	1.96	2.787(4)	166
O7H7*B*O6^ii^	0.85	2.10	2.883(5)	153

Symmetry codes: (i) 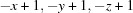; (ii) 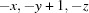.

## supplementary crystallographic information

### S1. Structural commentary

The title compound is isostructural with [M_2_(C_6_H_8_O_4_)(C_2_O_4_)_2_(H_2_O)_2_] [*M* = La, Sm, Gd] (Dan *et al.*, 2005; Li & Wang, 2010; Li, 2011). the asymmetric unit consists of one Dy^3+^ cation, a half of adipate anion, two half of oxalate anions and one aqua ligand (Fig. 1). The Dy atom is each coordinated by nine oxygen atoms, in which four oxygen atoms are from three adipate anions, four from two oxalate anions and one from a water molecule, to form a DyO_9_ polyhedron of a distorted tricapped trigonal-prismatic geometry. In the title complex, the adipate anions are located on a centre of symmetry and atom C3 is positionally disordered (C3A and C3B sites; occupancies 0.75/0.25). The adipate ligands act in a η^2^,µ_3_-η^2^,µ_3_-chelating-bridging o­cta­dentate coordination modes and link the DyO_9_ polyhedra into layers parallel to (010), in which the adjacent Dy···Dy distances are 4.20 (2) Å and 4.223 (9) Å, respectively. In the title complex, two symmetry independent oxalate ions are also located on centres of inversion and act as double bidentate (tetra­dentate) ligands in a zigzag chain along [001]. Through the oxalate and adipate ligands bridging inter­actions, the Dy atoms build up three-dimensional framework. The aqua ligand provides hydrogen-bond donors form hydrogen bonds with oxalate atoms O3 and O6.

The structure of the title complex is similar to that of other lanthanide (gadolinium, samarium and lanthanum) coordination polymers with adipate and oxalate ligands, and the mean Dy—O distance in the title complex of 2.438 Å is shorter than that of Ga—O (2.463 Å), Sm—O (2.482 Å) and La—O (2.566 Å).

### S2. Synthesis and crystallization

A mixture of DyCl_3_·6H_2_O(1.00 mmol, 0.38 g), oxalic acid (0.50 mmol, 0.05 g), adipic acid (0.50 mmol, 0.07 g), NaOH (2.00 mmol, 0.08 g) and H_2_O (10.0 ml) was heated in a 23 ml stainless steel reactor with a Teflon liner at 443 K for 48 h. A small amount of colorless plate-like crystals were filtered and washed with water and acetone. Yield 5% based on Dy.

### S3. Refinement

Crystal data, data collection and structure refinement details are summarized in Table 1. Atom C3 of the adipate anion is positionally disordered (C3A and C3B) and these atoms were refined with occupancies of 0.75/0.25. The C-bound H atoms were included in calculated positions and treated as riding atoms: C–H = 0.97 Å and *U*_iso_(H) = 1.2*U*_eq_(C)]. The water H-atoms were located in difference Fourier maps and were refined with distance restraints: O—H distance of 0.85 Å and *U*_iso_(H) = 1.5*U*_eq_(O). The highest density peak and deepest hole are located at 0.95 Å and 0.87 Å from the Dy atom, respectively.

### Crystal data

**Table:**

[Dy_2_(C_6_H_8_O_4_)(C_2_O_4_)_2_(H_2_O)_2_]	*Z* = 1
*M**_r_* = 681.20	*F*(000) = 316
Triclinic, *P*1	*D*_x_ = 3.138 Mg m^−^^3^
Hall symbol: -p 1	Mo *K*α radiation, λ = 0.71073 Å
*a* = 6.772 (2) Å	Cell parameters from 198 reflections
*b* = 6.929 (2) Å	θ = 4.6–28.3°
*c* = 8.949 (3) Å	µ = 10.37 mm^−^^1^
α = 104.916 (5)°	*T* = 295 K
β = 108.069 (4)°	Plate, colorless
γ = 104.306 (4)°	0.21 × 0.09 × 0.07 mm
*V* = 360.5 (2) Å^3^	

### Data collection

**Table:**

Bruker SMART APEXII CCD area-detector diffractometer	1234 independent reflections
Radiation source: fine-focus sealed tube	1179 reflections with *I* > 2σ(*I*)
Graphite monochromator	*R*_int_ = 0.014
φ and ω scans	θ_max_ = 25.0°, θ_min_ = 2.6°
Absorption correction: multi-scan (*SADABS*; Sheldrick, 2003)	*h* = −8→7
*T*_min_ = 0.245, *T*_max_ = 0.531	*k* = −6→8
1813 measured reflections	*l* = −10→7

### Refinement

**Table:**

Refinement on *F*^2^	Secondary atom site location: difference Fourier map
Least-squares matrix: full	Hydrogen site location: inferred from neighbouring sites
*R*[*F*^2^ > 2σ(*F*^2^)] = 0.021	H-atom parameters constrained
*wR*(*F*^2^) = 0.057	*w* = 1/[σ^2^(*F*_o_^2^) + (0.0422*P*)^2^ + 0.1716*P*] where *P* = (*F*_o_^2^ + 2*F*_c_^2^)/3
*S* = 1.05	(Δ/σ)_max_ < 0.001
1234 reflections	Δρ_max_ = 1.41 e Å^−^^3^
122 parameters	Δρ_min_ = −1.45 e Å^−^^3^
0 restraints	Extinction correction: *SHELXL97* (Sheldrick, 2008), Fc^*^=kFc[1+0.001xFc^2^λ^3^/sin(2θ)]^-1/4^
Primary atom site location: structure-invariant direct methods	Extinction coefficient: 0.0039 (11)

### Special details

**Table:**

Geometry. All e.s.d.'s (except the e.s.d. in the dihedral angle between two l.s. planes) are estimated using the full covariance matrix. The cell e.s.d.'s are taken into account individually in the estimation of e.s.d.'s in distances, angles and torsion angles; correlations between e.s.d.'s in cell parameters are only used when they are defined by crystal symmetry. An approximate (isotropic) treatment of cell e.s.d.'s is used for estimating e.s.d.'s involving l.s. planes.
Refinement. Refinement of *F*^2^ against ALL reflections. The weighted *R*-factor *wR* and goodness of fit *S* are based on *F*^2^, conventional *R*-factors *R* are based on *F*, with *F* set to zero for negative *F*^2^. The threshold expression of *F*^2^ > σ(*F*^2^) is used only for calculating *R*-factors(gt) *etc*. and is not relevant to the choice of reflections for refinement. *R*-factors based on *F*^2^ are statistically about twice as large as those based on *F*, and *R*- factors based on ALL data will be even larger.

### Fractional atomic coordinates and isotropic or equivalent isotropic displacement parameters (Å^2^)

**Table:**

	*x*	*y*	*z*	*U*_iso_*/*U*_eq_	Occ. (<1)
Dy	0.15576 (3)	0.34496 (3)	0.36280 (2)	0.01182 (14)	
O1	0.1519 (6)	0.5222 (6)	0.6470 (4)	0.0145 (7)	
O2	0.4801 (6)	0.5972 (6)	0.6395 (4)	0.0162 (8)	
O3	0.2699 (6)	0.1546 (6)	0.5391 (4)	0.0154 (8)	
O4	0.1535 (6)	−0.0812 (6)	0.6512 (5)	0.0181 (8)	
O5	−0.0072 (6)	0.2564 (6)	0.0712 (4)	0.0172 (8)	
O6	−0.1314 (7)	0.0203 (6)	−0.1913 (5)	0.0201 (8)	
O7	0.2677 (6)	0.6848 (6)	0.3381 (5)	0.0206 (8)	
H7A	0.4060	0.7539	0.3786	0.031*	
H7B	0.1863	0.7472	0.2931	0.031*	
C1	0.3632 (9)	0.6131 (8)	0.7234 (6)	0.0135 (11)	
C2	0.4635 (9)	0.7252 (9)	0.9114 (7)	0.0178 (12)	
H2A	0.3960	0.8298	0.9403	0.021*	
H2B	0.6211	0.8016	0.9479	0.021*	
C3A	0.4334 (12)	0.5740 (12)	1.0066 (9)	0.0162 (14)	0.75
H3A1	0.2770	0.4877	0.9624	0.019*	0.75
H3A2	0.4788	0.6577	1.1245	0.019*	0.75
C3B	0.576 (4)	0.593 (4)	1.005 (3)	0.0162 (14)	0.25
H3B1	0.6770	0.5536	0.9564	0.019*	0.25
H3B2	0.6640	0.6824	1.1227	0.019*	0.25
C4	0.1215 (8)	0.0215 (7)	0.5549 (6)	0.0127 (10)	
C5	−0.0395 (8)	0.0806 (8)	−0.0350 (7)	0.0150 (11)	

### Atomic displacement parameters (Å^2^)

**Table:**

	*U*^11^	*U*^22^	*U*^33^	*U*^12^	*U*^13^	*U*^23^
Dy	0.01277 (19)	0.01272 (18)	0.01252 (19)	0.00465 (11)	0.00650 (12)	0.00663 (11)
O1	0.0132 (17)	0.0169 (18)	0.0168 (18)	0.0054 (14)	0.0085 (15)	0.0081 (14)
O2	0.0179 (19)	0.0165 (19)	0.0181 (19)	0.0061 (15)	0.0117 (16)	0.0070 (14)
O3	0.0135 (18)	0.0175 (19)	0.0164 (18)	0.0052 (15)	0.0049 (15)	0.0093 (15)
O4	0.0134 (18)	0.022 (2)	0.0213 (19)	0.0068 (15)	0.0054 (15)	0.0131 (16)
O5	0.0220 (19)	0.0161 (19)	0.0167 (19)	0.0095 (15)	0.0092 (16)	0.0062 (15)
O6	0.027 (2)	0.022 (2)	0.014 (2)	0.0111 (17)	0.0083 (17)	0.0095 (16)
O7	0.0178 (19)	0.020 (2)	0.031 (2)	0.0080 (16)	0.0105 (17)	0.0177 (17)
C1	0.021 (3)	0.008 (2)	0.015 (3)	0.009 (2)	0.007 (2)	0.0074 (19)
C2	0.017 (3)	0.019 (3)	0.019 (3)	0.006 (2)	0.009 (2)	0.007 (2)
C3A	0.017 (3)	0.029 (4)	0.013 (3)	0.013 (3)	0.010 (3)	0.012 (3)
C3B	0.017 (3)	0.029 (4)	0.013 (3)	0.013 (3)	0.010 (3)	0.012 (3)
C4	0.018 (3)	0.008 (2)	0.015 (2)	0.0022 (19)	0.011 (2)	0.0058 (19)
C5	0.012 (2)	0.019 (3)	0.019 (3)	0.007 (2)	0.009 (2)	0.009 (2)

### Geometric parameters (Å, º)

**Table:**

Dy—O5	2.336 (4)	O7—H7A	0.8468
Dy—O4^i^	2.360 (4)	O7—H7B	0.8496
Dy—O3	2.376 (4)	C1—C2	1.501 (7)
Dy—O7	2.379 (4)	C2—C3A	1.525 (9)
Dy—O2^ii^	2.406 (3)	C2—C3B	1.57 (2)
Dy—O1^iii^	2.464 (4)	C2—H2A	0.9700
Dy—O6^iv^	2.535 (4)	C2—H2B	0.9700
Dy—O1	2.535 (3)	C3A—C3A^v^	1.529 (14)
Dy—O2	2.552 (4)	C3A—H3A1	0.9700
O1—C1	1.279 (7)	C3A—H3A2	0.9700
O2—C1	1.255 (6)	C3B—C3B^v^	1.40 (4)
O3—C4	1.256 (6)	C3B—H3B1	0.9700
O4—C4	1.253 (6)	C3B—H3B2	0.9700
O5—C5	1.256 (6)	C4—C4^i^	1.538 (10)
O6—C5	1.245 (7)	C5—C5^iv^	1.540 (11)
O6—Dy^iv^	2.535 (4)		
			
O5—Dy—O4^i^	90.45 (13)	C1—O1—Dy	95.2 (3)
O5—Dy—O3	134.27 (12)	Dy^iii^—O1—Dy	115.28 (14)
O4^i^—Dy—O3	68.93 (12)	C1—O2—Dy^ii^	148.4 (3)
O5—Dy—O7	78.20 (13)	C1—O2—Dy	95.1 (3)
O4^i^—Dy—O7	142.35 (13)	Dy^ii^—O2—Dy	115.66 (13)
O3—Dy—O7	141.46 (12)	C4—O3—Dy	117.8 (3)
O5—Dy—O2^ii^	91.79 (12)	C4—O4—Dy^i^	118.7 (3)
O4^i^—Dy—O2^ii^	142.20 (13)	C5—O5—Dy	123.8 (3)
O3—Dy—O2^ii^	82.95 (12)	C5—O6—Dy^iv^	117.5 (3)
O7—Dy—O2^ii^	74.65 (13)	Dy—O7—H7A	116.9
O5—Dy—O1^iii^	82.15 (12)	Dy—O7—H7B	128.4
O4^i^—Dy—O1^iii^	69.07 (12)	H7A—O7—H7B	114.8
O3—Dy—O1^iii^	122.91 (11)	O2—C1—O1	118.8 (5)
O7—Dy—O1^iii^	73.84 (12)	O2—C1—C2	122.2 (5)
O2^ii^—Dy—O1^iii^	148.49 (12)	O1—C1—C2	119.0 (4)
O5—Dy—O6^iv^	65.84 (12)	O2—C1—Dy	59.8 (3)
O4^i^—Dy—O6^iv^	70.76 (13)	O1—C1—Dy	59.1 (2)
O3—Dy—O6^iv^	68.84 (12)	C2—C1—Dy	173.1 (4)
O7—Dy—O6^iv^	132.07 (13)	C1—C2—C3A	113.3 (5)
O2^ii^—Dy—O6^iv^	75.88 (12)	C1—C2—C3B	111.8 (9)
O1^iii^—Dy—O6^iv^	127.55 (12)	C1—C2—H2A	108.9
O5—Dy—O1	146.78 (12)	C3A—C2—H2A	108.9
O4^i^—Dy—O1	80.36 (12)	C3B—C2—H2A	134.9
O3—Dy—O1	71.64 (12)	C1—C2—H2B	108.9
O7—Dy—O1	89.76 (13)	C3A—C2—H2B	108.9
O2^ii^—Dy—O1	114.93 (11)	C3B—C2—H2B	76.5
O1^iii^—Dy—O1	64.72 (14)	H2A—C2—H2B	107.7
O6^iv^—Dy—O1	137.19 (12)	C2—C3A—C3A^v^	113.2 (7)
O5—Dy—O2	146.25 (12)	C2—C3A—H3A1	108.9
O4^i^—Dy—O2	123.15 (12)	C3A^v^—C3A—H3A1	108.9
O3—Dy—O2	69.30 (12)	C2—C3A—H3A2	108.9
O7—Dy—O2	72.79 (13)	C3A^v^—C3A—H3A2	108.9
O2^ii^—Dy—O2	64.34 (13)	H3A1—C3A—H3A2	107.8
O1^iii^—Dy—O2	105.43 (11)	C3B^v^—C3B—C2	113 (2)
O6^iv^—Dy—O2	124.52 (12)	C3B^v^—C3B—H3B1	108.9
O1—Dy—O2	50.76 (11)	C2—C3B—H3B1	108.9
O5—Dy—C1	158.48 (13)	C3B^v^—C3B—H3B2	108.9
O4^i^—Dy—C1	101.76 (14)	C2—C3B—H3B2	108.9
O3—Dy—C1	67.18 (13)	H3B1—C3B—H3B2	107.7
O7—Dy—C1	81.38 (14)	O4—C4—O3	125.9 (5)
O2^ii^—Dy—C1	89.29 (13)	O4—C4—C4^i^	116.9 (5)
O1^iii^—Dy—C1	85.74 (13)	O3—C4—C4^i^	117.2 (5)
O6^iv^—Dy—C1	134.90 (13)	O6—C5—O5	127.2 (5)
O1—Dy—C1	25.66 (13)	O6—C5—C5^iv^	116.0 (6)
O2—Dy—C1	25.14 (13)	O5—C5—C5^iv^	116.8 (6)
C1—O1—Dy^iii^	132.5 (3)		
			
O5—Dy—O1—C1	−138.0 (3)	O1^iii^—Dy—O5—C5	−135.5 (4)
O4^i^—Dy—O1—C1	146.2 (3)	O6^iv^—Dy—O5—C5	1.9 (4)
O3—Dy—O1—C1	75.4 (3)	O1—Dy—O5—C5	−139.6 (4)
O7—Dy—O1—C1	−70.3 (3)	O2—Dy—O5—C5	118.4 (4)
O2^ii^—Dy—O1—C1	2.6 (3)	C1—Dy—O5—C5	168.2 (4)
O1^iii^—Dy—O1—C1	−142.5 (4)	Dy^ii^—O2—C1—O1	−171.1 (4)
O6^iv^—Dy—O1—C1	98.7 (3)	Dy—O2—C1—O1	−4.5 (5)
O2—Dy—O1—C1	−2.5 (3)	Dy^ii^—O2—C1—C2	5.7 (9)
O5—Dy—O1—Dy^iii^	4.5 (3)	Dy—O2—C1—C2	172.3 (4)
O4^i^—Dy—O1—Dy^iii^	−71.31 (15)	Dy^ii^—O2—C1—Dy	−166.6 (6)
O3—Dy—O1—Dy^iii^	−142.17 (17)	Dy^iii^—O1—C1—O2	−127.1 (4)
O7—Dy—O1—Dy^iii^	72.19 (15)	Dy—O1—C1—O2	4.5 (5)
O2^ii^—Dy—O1—Dy^iii^	145.10 (14)	Dy^iii^—O1—C1—C2	56.0 (6)
O1^iii^—Dy—O1—Dy^iii^	0.0	Dy—O1—C1—C2	−172.3 (4)
O6^iv^—Dy—O1—Dy^iii^	−118.82 (18)	Dy^iii^—O1—C1—Dy	−131.7 (4)
O2—Dy—O1—Dy^iii^	139.9 (2)	O5—Dy—C1—O2	−86.1 (5)
C1—Dy—O1—Dy^iii^	142.5 (4)	O4^i^—Dy—C1—O2	150.6 (3)
O5—Dy—O2—C1	138.8 (3)	O3—Dy—C1—O2	89.7 (3)
O4^i^—Dy—O2—C1	−35.1 (3)	O7—Dy—C1—O2	−67.6 (3)
O3—Dy—O2—C1	−80.2 (3)	O2^ii^—Dy—C1—O2	7.0 (3)
O7—Dy—O2—C1	106.9 (3)	O1^iii^—Dy—C1—O2	−141.9 (3)
O2^ii^—Dy—O2—C1	−172.2 (4)	O6^iv^—Dy—C1—O2	76.1 (3)
O1^iii^—Dy—O2—C1	39.7 (3)	O1—Dy—C1—O2	−175.4 (5)
O6^iv^—Dy—O2—C1	−123.4 (3)	O5—Dy—C1—O1	89.3 (5)
O1—Dy—O2—C1	2.6 (3)	O4^i^—Dy—C1—O1	−34.1 (3)
O5—Dy—O2—Dy^ii^	−49.0 (3)	O3—Dy—C1—O1	−95.0 (3)
O4^i^—Dy—O2—Dy^ii^	137.16 (15)	O7—Dy—C1—O1	107.8 (3)
O3—Dy—O2—Dy^ii^	92.06 (16)	O2^ii^—Dy—C1—O1	−177.6 (3)
O7—Dy—O2—Dy^ii^	−80.87 (16)	O1^iii^—Dy—C1—O1	33.5 (3)
O2^ii^—Dy—O2—Dy^ii^	0.001 (1)	O6^iv^—Dy—C1—O1	−108.5 (3)
O1^iii^—Dy—O2—Dy^ii^	−148.06 (15)	O2—Dy—C1—O1	175.4 (5)
O6^iv^—Dy—O2—Dy^ii^	48.8 (2)	O2—C1—C2—C3A	−110.4 (6)
O1—Dy—O2—Dy^ii^	174.8 (2)	O1—C1—C2—C3A	66.3 (7)
C1—Dy—O2—Dy^ii^	172.2 (4)	O2—C1—C2—C3B	−71.7 (11)
O5—Dy—O3—C4	−74.6 (4)	O1—C1—C2—C3B	105.1 (10)
O4^i^—Dy—O3—C4	−5.9 (3)	C1—C2—C3A—C3A^v^	68.2 (9)
O7—Dy—O3—C4	145.5 (3)	C3B—C2—C3A—C3A^v^	−27.2 (14)
O2^ii^—Dy—O3—C4	−160.2 (3)	C1—C2—C3B—C3B^v^	−70 (2)
O1^iii^—Dy—O3—C4	39.2 (4)	C3A—C2—C3B—C3B^v^	29.9 (15)
O6^iv^—Dy—O3—C4	−82.6 (3)	Dy^i^—O4—C4—O3	−174.4 (4)
O1—Dy—O3—C4	80.6 (3)	Dy^i^—O4—C4—C4^i^	4.9 (7)
O2—Dy—O3—C4	134.6 (4)	Dy—O3—C4—O4	−175.0 (4)
C1—Dy—O3—C4	107.6 (4)	Dy—O3—C4—C4^i^	5.8 (7)
O4^i^—Dy—O5—C5	−66.7 (4)	Dy^iv^—O6—C5—O5	178.5 (4)
O3—Dy—O5—C5	−6.3 (5)	Dy^iv^—O6—C5—C5^iv^	−2.6 (7)
O7—Dy—O5—C5	149.5 (4)	Dy—O5—C5—O6	177.5 (4)
O2^ii^—Dy—O5—C5	75.6 (4)	Dy—O5—C5—C5^iv^	−1.4 (7)

Symmetry codes: (i) −*x*, −*y*, −*z*+1; (ii) −*x*+1, −*y*+1, −*z*+1; (iii) −*x*, −*y*+1, −*z*+1; (iv) −*x*, −*y*, −*z*; (v) −*x*+1, −*y*+1, −*z*+2.

### Hydrogen-bond geometry (Å, º)

**Table:**

*D*—H···*A*	*D*—H	H···*A*	*D*···*A*	*D*—H···*A*
O7—H7*A*···O3^ii^	0.85	1.96	2.787 (4)	166
O7—H7*B*···O6^vi^	0.85	2.10	2.883 (5)	153

Symmetry codes: (ii) −*x*+1, −*y*+1, −*z*+1; (vi) −*x*, −*y*+1, −*z*.
